# 3D evaluation of the extracellular matrix of hypoxic pancreatic islets using light sheet fluorescence microscopy

**DOI:** 10.1080/19382014.2023.2298518

**Published:** 2024-01-24

**Authors:** Matias Ramirez, Estelle Bastien, Heeyoung Chae, Pierre Gianello, Patrick Gilon, Caroline Bouzin

**Affiliations:** a Pole of Experimental Surgery and Transplantation, Institute of Experimental and Clinical Research, Université catholique de Louvain, Brussels, Belgium; b Pole of Pharmacology and Therapeutics, Institute of Experimental and Clinical Research, Université catholique de Louvain, Brussels, Belgium; c Pole of Endocrinology, Diabetes and Nutrition, Institute of Experimental and Clinical Research, Université catholique de Louvain, Brussels, Belgium; d Laboratory of Experimental Surgery and Transplantation, Institute of Experimental and Clinical Research, Université catholique de Louvain, Brussels, Belgium; e Institute of Experimental and Clinical Research, Université catholique de Louvain, Brussels, Brussels, Belgium

**Keywords:** 3D microscopy, extracellular matrix, hypoxia, light sheet fluorescence microscopy, pancreatic islets, tissue clearing

## Abstract

Pancreatic islet transplantation is a promising treatment for type 1 diabetes, but the survival and function of transplanted islets are hindered by the loss of extracellular matrix (ECM) during islet isolation and by low oxygenation upon implantation. This study aimed to evaluate the impact of hypoxia on ECM using a cutting-edge imaging approach based on tissue clearing and 3D microscopy. Human and rat islets were cultured under normoxic (O_2_ 21%) or hypoxic (O_2_ 1%) conditions. Immunofluorescence staining targeting insulin, glucagon, CA9 (a hypoxia marker), ECM proteins (collagen 4, fibronectin, laminin), and E-cadherin (intercellular adhesion protein) was performed on fixed whole islets. The cleared islets were imaged using Light Sheet Fluorescence Microscopy (LSFM) and digitally analyzed. The volumetric analysis of target proteins did not show significant differences in abundance between the experimental groups. However, 3D projections revealed distinct morphological features that differentiated normoxic and hypoxic islets. Under normoxic conditions, ECM could be found throughout the islets. Hypoxic islets exhibited areas of scattered nuclei and central clusters of ECM proteins, indicating central necrosis. E-cadherin was absent in these areas. Our results, demonstrating a diminution of islets’ functional mass in hypoxia, align with the functional decline observed in transplanted islets experiencing low oxygenation after grafting. This study provides a methodology combining tissue clearing, multiplex immunofluorescence, Light Sheet Fluorescence Microscopy, and digital image analysis to investigate pancreatic islet morphology. This 3D approach allowed us to highlight ECM organizational changes during hypoxia from a morphological perspective.

## GRAPHICAL ABSTRACT

**Figure:**
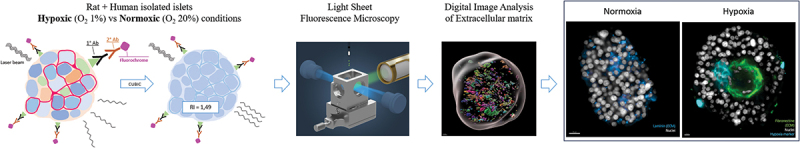

## Introduction

Type 1 Diabetes Mellitus (T1D) is a disease that affects more than eight million people worldwide and is a leading cause of morbidity in developed countries.^1^ T1D has a complex etiology and is characterized by a lack of endogenous production of insulin.^2,3^ It is normally treated with the daily administration of insulin via injections or pumps.^4^

A promising alternative is the transplantation of pancreatic islets of Langerhans.^5,6^ Several studies have demonstrated the capacity of grafted islets to regulate blood glucose physiologically.^5,7^ However, this procedure is limited to a handful of centers worldwide and is clinically applied under very specific criteria, due to still suboptimal graft survival, necessitating 2-to-3 donors per recipient.

To be transplanted, islets are isolated from human cadaveric pancreas. Isolation and transplantation induce a series of processes that compromise islets’ survival and function. Among them, cell-ECM detachment and hypoxia play a predominant role.^8,9^

During isolation, most of the islets’ native extracellular matrix (ECM) is lost due to enzymatic digestion, triggering a process of cellular apoptosis named anoikis.^10,11^ The mechanisms enrolled in anoikis have been largely reported, demonstrating that the scarcity of ECM triggers (a) compensatory mechanisms, e.g., the overexpression of E-Cadherin, reinforcing cell-to-cell adhesion, and (b) a series of molecular pathways resulting in apoptosis.^12–15^

Islets’ ECM is composed of many proteins, of which Collagen 4, Fibronectin, and Laminin have been identified as forming the basal membrane (BM), a capsule that surrounds islets and that is lost during isolation, with Collagen 4 and Laminin also coating the islet’s inner microvasculature.^10,14,16–18^ Conversely, ECM supplementation after isolation reduces cytokine stress and fibrosis and overall enhances graft survival and long-term insulin production.^10,13,16,19–24^

Another issue to overcome for optimal islet transplantation is oxygenation. Isolation destroys the islets’ dedicated blood supply.^25^ Upon implantation, islets suffer from hypoxic conditions until a new microvasculature is developed around them.^26,27^ Islets’ oxygenation, relying on O_2_ diffusion, varies between the implantation sites but has been consistently reported as being under the islets’ physiological needs.^27^ Hypoxia has been shown to decrease insulin secretion and to induce several forms of cell death, from necrosis to apoptosis.^28,29^

Interestingly, hypoxia and cell-ECM detachment trigger similar molecular mechanisms: in a dialog of activation and inhibition of regulators such as the MAPK cascade, p38, JNK, and ERK, both processes lead to the induction of cytokine secretion, which in turn intensifies death mechanisms, as well as the dysregulation of the Bcl-2 family leading to apoptosis.^9,13,16,30,31^ Thus, ECM attachment and oxygenation are both determinants of islet survival and function.

Little is known about the modifications of islets’ ECM after isolation, especially under hypoxic conditions.^9,32^ We hypothesize that hypoxia hinders remodeling, as this process necessitates active secretion of proteins. Therefore, we have developed a protocol of 3D imaging to evaluate the influence of hypoxia on isolated islets’ ECM remodeling.

A 3D approach allows for both protein quantification and qualitative evaluation. 3D imaging improves visualization of the islet architecture, permitting a better analysis of the distribution of cells and extracellular matrix.^33,34^ Recent developments in light sheet microscopy and tissue clearing have made possible the efficient imaging of whole organs or organ subunits, like islets.^35,36^ Light sheet fluorescence microscopy (LSFM) is a technique that uses a single plane (sheet of light) illumination, allowing fast, high-resolution, multichannel imaging of thick samples.^37^ To reach 3D imaging without signal loss in depth, tissues need to be transparent, even for small, dense structures like pancreatic islets (ø 50–250 µm). This is achieved using clearing techniques that homogenize the refractive index of the different structures of the organ, thus reducing diffraction and light absorption while preserving shape and immunofluorescence.^35^

Our study is a proof of concept, demonstrating the feasibility of a LSFM-based approach to isolated islet evaluation, and highlights the importance of an efficient 3D approach. We explored the influence of hypoxia on ECM remodeling, providing morphological evidence of ECM changes in islets in the context of transplantation. To the best of our knowledge, we are the first to validate such a process applied to isolated islets and to address the effects of hypoxia on the ECM from a 3D perspective.^38^

## Materials and methods

### Islet procurement

*Rat islets*. All protocols were approved by the Ethical Committee [2020/UCL/MD/016]. Male Wistar rats were put under inhalatory anesthesia and euthanized by exsanguination after laparotomy. 10 ml of Collagenase P solution (1 U/ml – Roche, #11213873001) was injected into the pancreas through the bile duct, under direct observation, to ensure homogeneous distribution throughout the organ. The pancreas was then dissected free and stored in ice-cold Krebs awaiting processing.

Digestion was done by putting the pancreas at 37°C in a water bath for 15 minutes. Digestion was controlled by direct observation and was stopped by adding cold Krebs, followed by 3–4 washes. Islets were then handpicked under a dissection microscope.

*Human islets* were procured at Tebubio. Two batches were used in our study: 58 years old, BMI 29, HbA1c 5.0%, and 32 years old, BMI 26.9, HbA1c 4.9%. After reception, they were cultured for 48 hours, as instructed by the provider, to allow for recovery.

### Experimental workflow

After isolation, islets were divided into three groups of approximately 400 islets and cultured in RPMI (Invitrogen, #11875093) supplemented with fetal bovine serum 10% (Lonza, #26140079), penicillin-streptomycin 100 U/ml (Lonza, #09-757F), glutamine 2 mM (Lonza, #BE17-605E), and 10 mM or 5 mM glucose for rat and human islets, respectively. Islets of the first group were harvested after overnight culture in normoxic (O2 21%, CO2 5%) conditions (Normoxia Day 1 (ND1)). Islets from the two other groups were cultured for a total of 5 days either in normoxic or hypoxic (O2 1%, CO2 5%, AirLiquide, mix #23209469–10) conditions (Normoxia Day 5 (ND5) and Hypoxia Day 5 (HD5)). Hypoxia was reached by placing the culture dishes in a hypoxic chamber (StemCell, #27310).

### Viability

An islet sample was taken from culture dishes, centrifuged, and incubated in 200 µl of 1% fluorescein (Thermo Fisher, #119241000) and 1% propidium iodide (AcrosOrganics, #440301000) in PBS for 5 minutes, then rinsed for 10 minutes in PBS. Islets were placed in culture dishes (Greiner, Cellview, #627871) and imaged using an epifluorescence microscope (Zeiss AxioObserver.z1). Images were processed with QuPath software (v0.3.0) using an ad hoc protocol to measure the stained areas in the green (viable cells) and red (dead cells) channels.^39^ Viability was calculated as the ratio between the green area over the area of the combined channels.

### Functional assessment

Glucose-stimulated insulin secretion (GSIS) tests were performed with batches of 3–4 islets. Hand-picked islets (similar size) were incubated at 37°C for 1 hour, in 1 mL Krebs buffer containing glucose either 1 mM or 15 mM. At the end of the incubation, the culture supernatant was collected for measurement of insulin (homemade assay^40^ and glucagon (Merck Millipore, Burlington, MA), and islets were collected and sonicated in 10 mmol/l Tris, 0.2 mol/l NaCl and 10 mmol/l EDTA for measurement of their DNA, insulin, and glucagon content. All secretion experiments were carried out in duplicate.

### Fixation and storage

After culture, islets were washed three times with PBS, fixed using 4% formaldehyde supplemented with 0.1% Triton X-100 (Sigma, #T9284) for 3 h, followed by three washes of 5 minutes using PBS. They were then stored at 4°C in a solution of PBS, 0.01% formaldehyde, and 0.005% sodium azide until further processing (maximum 1 month).

### Antibodies validation

All antibodies used are listed in Extended Data – Table S1 – Antibodies.

#### Absence of cross-reactivity of secondary antibodies

Fixed islets were embedded in 1% agarose and then included in paraffin. Sections of 5 µm were cut using a microtome.

2D sections of islets were incubated with each primary antibody individually, which was then revealed either with the adequate fluorochrome-conjugated secondary antibody or with a mix of all secondary antibodies. Hoechst was used for nuclear staining.

#### Antibody penetration

Islets were treated as described in the 3D IF section, up to finalizing the primary antibody step. Islets were then fixed using formaldehyde 4% for 1 hour at RT, included, and cut. Then, sections were either (a) incubated with anti-mouse and anti-rabbit secondary antibodies for 1 hour at RT, together with Hoechst for nuclear staining, or (b) followed standard IF staining (primary and secondary antibodies, as described before).

Representative images of the validation tests can be found in the Extended Data – Figure S1.

### 3D immunofluorescence

Unless otherwise stated, all steps were performed in 24-well plates using volumes of 1 ml under agitation at room temperature. Rinsing was done by washing three times for 5 minutes.

Fixed islets were washed with 0.05% Triton-X100 in PBS, followed by a permeabilization step in 0.1% Triton-X100 in PBS for 1 h. Unspecific binding block was done with a 3 hours incubation in 5% BSA (Carl Roth, #3854.3), 0.1% Triton-X100, and 0.01% sodium azide in PBS. Primary and secondary antibodies were incubated in a volume of 500 µl at 37°C under agitation in PBS containing 1% BSA, 0.1% Triton-X100, and 0.01% sodium azide. Antibodies (primaries and secondaries) were incubated for 3 or 7 days each, respectively for human and rat islets, with three washing steps of 20 minutes in between. Finally, islets were washed and fixed for 1 hour in formaldehyde 4%. Antibodies are listed in Extended Data – Table S1.

### Clearing and embedding

Islets were cleared by placing them in 1 ml of CUBIC1 solution (Extended Data – Table S2) for 24 hours.^36^ After washing with PBS, islets were handpicked to eliminate as much supernatant as possible, and placed in a well with 1 ml of 1% agarose low melting point (Sigma, #39346-81-1) and kept at 56°C. Islets were gently aspirated using a capillary (ø 1 mm) and left at room temperature for 2 hours to solidify. The agarose rods were then de-molded and incubated for 4 hours at 37°C in a solution of CUBIC2 (Extended Data – Table S2) diluted 1:1 in water. Finally, samples were incubated overnight under agitation at 37°C in 100% CUBIC 2 (refractive index = 1.4929 at room temperature) containing 1 µg/ml Hoechst 3342 (Invitrogen® #H3570) for nuclear staining.

### Light sheet fluorescence microscopy (LSFM)

The agarose rods were aspirated into a capillary (ø 1 mm), which was placed on an adequate sample holder and introduced in the chamber of the LSFM (Zeiss Lightsheet.z1) filled with CUBIC2. For imaging, the tip of the agarose rod containing the islets was pushed out of the capillary. Acquisition parameters were kept constant for all samples.

Acquisition parameters are summarized in Extended Data – Table S3 - Confocal vs LSFM.

For rat islets, acquisitions were performed with 5×/0.1 illumination and 5×/0.16 detection objectives, using dual side illumination with pivot scan.

To be able to image ECM details, human islets were imaged using 10×/0.2 illumination objectives and a 20×/1.0 detection objective.

### Laser scanning confocal microscopy

After LSFM imaging, an agarose rod containing islets was placed in a dish with a coverslip bottom and imaged with a Zeiss LSM800 microscope using a 20×/0.8 Plan-Apochromat objective.

### Image processing

The two image sets generated using dual-side acquisition were fused into one image, using a discrete Fourier transformation with Zeiss Zen Black Edition (version 9.2.8.54).

### Image analysis

Images were converted using Bitplane’s Imaris Batch Converter and imported into Imaris Arena. Imaris (v9.8) was used to produce orthogonal and 3D projections to evaluate islets macroscopically and to perform quantifications. Each islet was treated individually.

For quantitative analyses, each islet was first delineated in 3D using a surface wizard, applying a threshold to the sum of the pixel values of all channels smoothed with a Gaussian blur. When needed, artifacts were manually discarded, and islets were separated if they coexisted in the same image.

Target protein expression was quantified using an ad-hoc protocol provided in the Extended data – DIA protocols. Briefly, it consisted of a pre-processing step using a Gaussian filter and a detection based on a threshold using either absolute intensity or background subtraction (depending on the protein). No post-processing filters were applied. Results were expressed as the percentage of stained volume over the islet volume.

### Statistics

All calculations were computed using IBM SPSS (v25). Datasets were evaluated using Q-Q plots and the Shapiro-Wilk test; if necessary, transformations were performed to ensure normality. Results are presented as mean ± SD of non-transformed data.

Correlations were evaluated using Pearson’s Correlation Coefficient and means comparisons were done in a two-by-two independent means fashion. ANOVA was used to evaluate the influence of condition on islet volume.

## Results

### Study design

**Figure 1.**
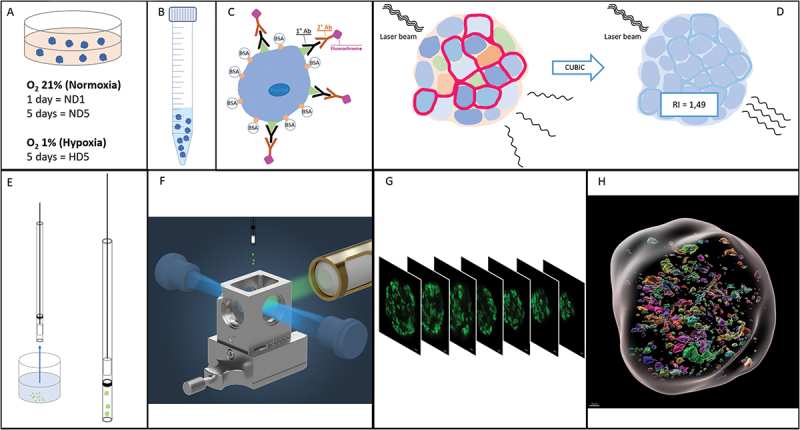
Workflow of the study. (a) Islet culture in different oxygenation conditions. (b) Fixation and storage. (c) Immunofluorescence:: permeabilization, unspecific site blockage, primary and secondary antibodies incubation (3 and 7 days each, for human and rat islets, respectively). (d) Using the CUBIC approach, refractive indexes homogenization. (e) Embedding of fluorescent and transparent islets in agarose and aspiration in a capillary. (f) Positioning of the islets, in an agarose rod, inside the imaging chamber of the LSFM (adapted from Zeiss® z.1 user manual). (g) Imaging of sequential virtual sections. (h) Image processing: 3D region of interest (gray) containing volumes stained for fibronectin.

This study is divided into two parts: (a) the technical validation of the methodology (immunofluorescence, clearing, LSFM, and digital image analysis), using rat and human islets, and (b) the evaluation of the effect of hypoxia on target proteins abundance and distribution in human islets. Figure 1 summarizes the methodology workflow.

For both parts, we have compared three experimental groups: islets cultured in normal conditions for one day (ND1, Normoxia Day 1), and islets subjected to five days of either normal or hypoxic culture conditions (ND5, Normoxia Day 5, and HD5, Hypoxia Day 5).

In the technical part, we validated hypoxic conditions, primary and secondary antibodies, and we correlated the digital image analysis (DIA) quantification of insulin and glucagon to gold standard measurements using radioimmunoassay (RIA).

We then applied our validated methodology to morphologically evaluate and quantify Collagen 4, Fibronectin, Laminin, and E-Cadherin in individual islets.

### Light sheet fluorescence microscopy provides high-quality 3D images of islets

**Figure 2.**
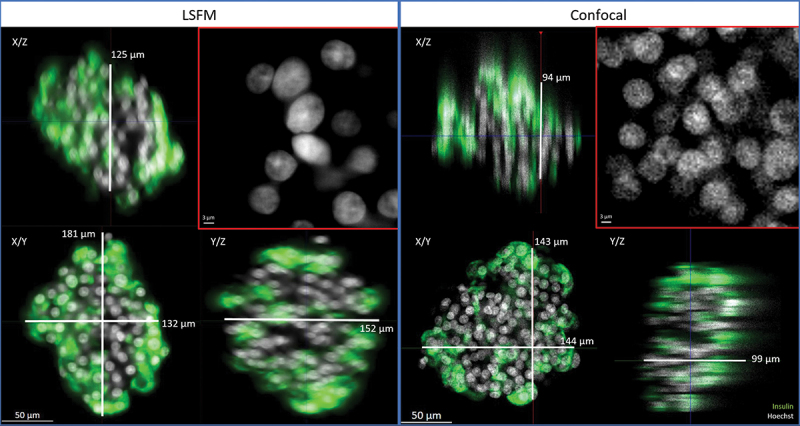
Comparison of imaging quality using LSFM and confocal microscopies. The same human islet was imaged with both microscopes, with if for insulin (green) and nuclei (Hoechst, white). The position varies as it is impossible to acquire systematically from the same angle with two different microscopes. Bottom left of each data set: 2D cross-section in the middle of the islet (X/Y view). Top-left and bottom right: X/Z and Y/Z orthogonal projections. Top right (red square): magnification of Hoechst-labeled nuclei in X/Y. Image resolution was superior in confocal microscopy, with overall crispier staining. However, this was detrimental to proper nuclear segmentation in DIA, requiring additional pre-processing steps. Orthogonal projections highlight the flattening of the sample in z and spherical aberrations when using confocal microscopy.

Among the available 3D imaging technologies, we compared confocal laser scanning microscopy and LSFM acquisitions of the same immunostained and cleared human islet (ø 180 µm). On both microscopes, z-stacks were acquired sequentially for each of the four fluorescence channels with a 20× detection objective (specifications are summarized in Extended Data – Table S3). The whole, full-depth islet was imaged. The comparison is illustrated in Figure 2.

Sample transparency was verified with LSFM and confocal microscopy: sharp staining with preserved fluorescence intensity was observed throughout the whole sample (in XY and in Z).

We identified several advantages of the LSFM over confocal acquisitions. First, imaging was much faster with LSFM (3.5 min per islet vs. 31 min with confocal), mainly owing to the detection system (camera vs. detector). Secondly, as illustrated in Figure 2, although optimal step size was applied on both systems (according to Nyquist criteria), axial resolution was superior with LSFM. Indeed, 3D projections of confocal images displayed a flattened appearance, while similar islet diameters were measured laterally and axially on LSFM images without any image processing. Thirdly, confocal images were edgier but more speckled, especially for Hoechst-stained nuclei. This complicates further segmentation in the DIA process. The resolution of LSFM images allowed for visualization of subcellular structures such as nuclei, cell membranes, and insulin granules.

Taken together, our observations validate the clearing methodology and highlight LSFM as the most efficient whole islet imaging technology.

### Hypoxia hinders islets’ survival and function – validation of the hypoxic experimental model

**Figure 3.**
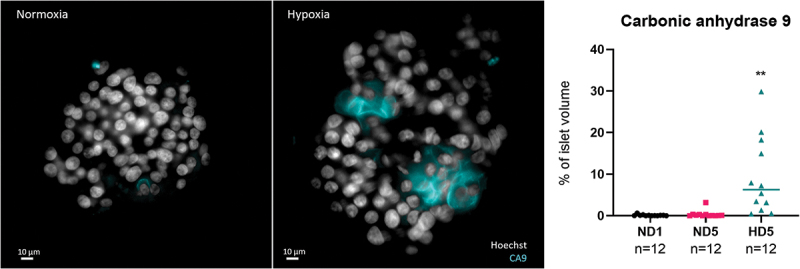
Left and central panels: representative 2D cross-sections in the middle of the human islets, with if for CA9 and Hoechst. CA9 is a trans-membrane protein, used here as a marker of hypoxia. Its presence after 5 days in normal culture conditions was sporadic (left), while under hypoxic conditions (group HD5, right), it could be found in all islets, expressed in large areas. Note the alteration in nuclear shape in areas around hypoxia. Right panel: quantification of CA9, expressed as percentage of total islet volume, showing significant presence of CA9 after 5 days in hypoxia.

To validate the effect of hypo-oxygenation, rat and human islets were cultured in hypoxic conditions using a hypoxia chamber with O_2_ at 1% for 5 days (HD5) and compared to islets cultured in normoxic conditions for 1 or 5 days (ND1, ND5). Immunofluorescence for Carbonic Anhydrase 9 (CA9) was used to identify hypoxia (Figure 3). CA9 is part of the hypoxia cascade and a more stable downstream target of HIF-1.^29,41^ As summarized in the graph of Figure 3, CA9 was present predominantly in the hypoxia group, although sporadic CA9-positive cells were identified in the ND1 and ND5. There were no statistically significant differences between the normoxia groups, while a significant difference was observed between HD5 and ND1 (*p* = .006) and ND5 (*p* = .007).

After 5 days in culture, viability was, respectively for normal and hypoxic conditions, 88% and 62% in human islets (*p* = .04), and 79% and 29% in rat islets (*p* = .001). Overall, this shows that hypoxia diminishes islet survival.

Rat islets were then incubated with 1 mM (G1) or 15 mM (G15) of glucose to assess their functionality. Insulin secretion rose from 1.9 (G1) to 7.5 (G15) ng/hour/islet for ND1 and from 2.6 (G1) to 3.6 (G15) ng/hour/islet for ND5 and dropped to 0.2 (G1) to 0.3 (G15) ng/hour/islet for HD5, *n* = 12 per condition, *p* < .01. A graphic illustrating these differences can be found in the Extended Data – Figure S2. These results show that glucose-stimulated insulin secretion diminishes at ND5 and is virtually stopped at HD5, showing the detrimental effect of hypoxia on glucose-stimulated insulin secretion.

### Insulin and glucagon quantification: digital image analysis (DIA) produces similar results to radioimmunoassay (RIA)

To validate immunostaining quantification on 3D images by DIA, we compared the impact of hypoxia on insulin and glucagon contents measured by DIA and by RIA. Measurements were performed on different rat islets from the same batch. DIA quantification generated volumetric values normalized to islet volume, while protein weight was measured by RIA and normalized to DNA content.

**Figure 4.**
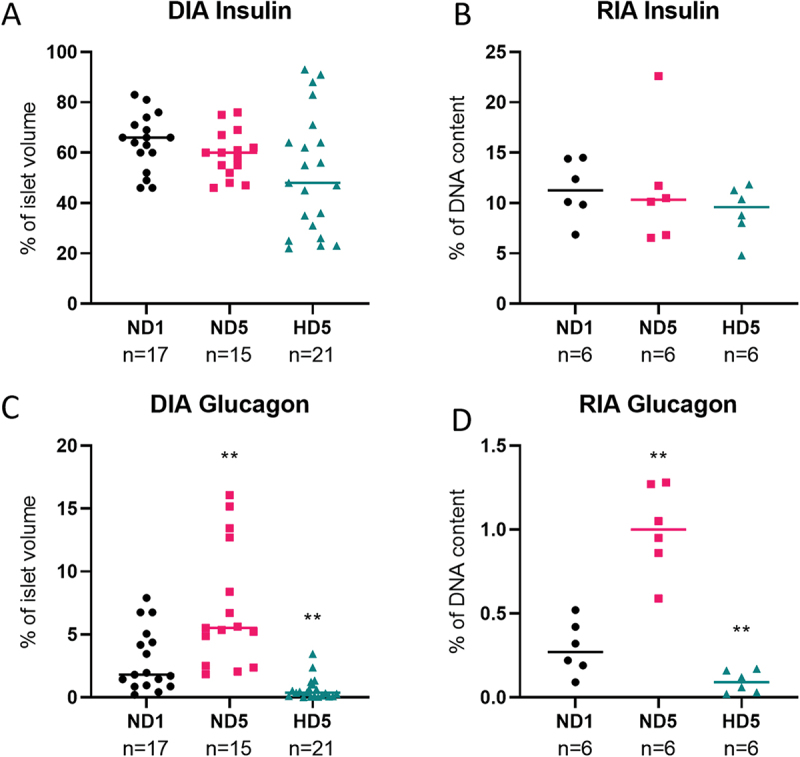
Comparison of the quantification of rat islets’ insulin content with two different techniques. (a and c) percentage of volume occupied by insulin or glucagon inside the islet, determined by Digital Image Analysis. (b and d) weight of these proteins measured by radioimmunoassay, expressed as a percentage of DNA content.

Figure 4 illustrates that both techniques showed consistency between DIA and RIA measurements, notably the maintenance of insulin content under hypoxia, and an increase or decrease of glucagon abundance, respectively in ND5 and HD5 conditions (*p* < .01).

Both DIA and RIA depict similar data distribution.

N represents the number of islets.

*3D quantitative evaluation of islets and ECM components*

Human islets were used to address changes in the ECM during hypoxia. To ensure that islet size did not bias our analysis, we confirmed using ANOVA that culture conditions did not influence islet size (*p* = .277). Furthermore, the influence of size on specific cues was evaluated. They will be mentioned only if they are significant.

The effects of hypoxia on Collagen 4, Fibronectin, Laminin, and E-Cadherin were evaluated on human islets. Insulin and glucagon staining allowed islet identification, thus avoiding the bias of analyzing non-endocrine islet cells.

**Figure 5.**
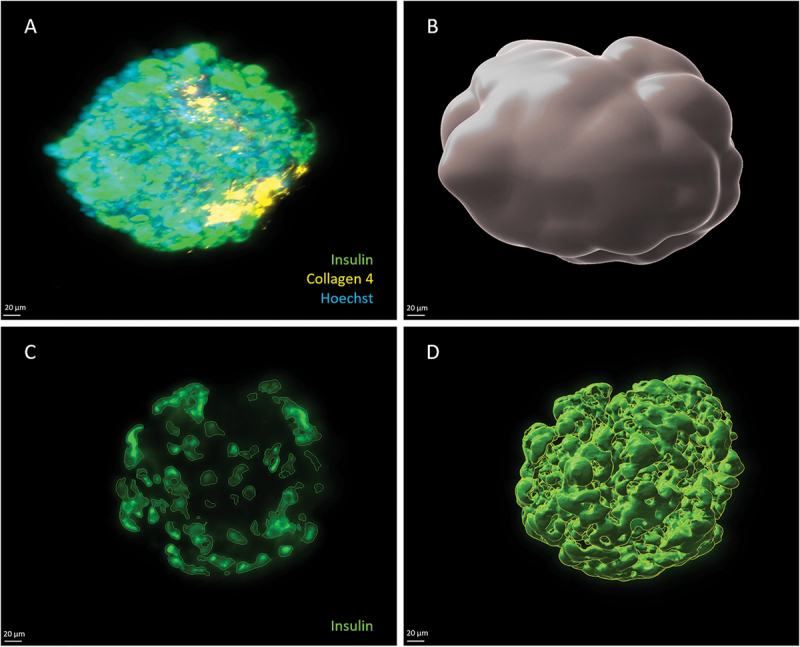
Digital Image Analysis process. (a) 3D projection of an islet before processing; (b) 3D projection of a region of interest, used to calculate total islet volume. (c) 2D projection showing target protein (insulin) identified using a standardized intensity threshold; (d) 3D projection of the target protein stained volume.

**Figure 6.**
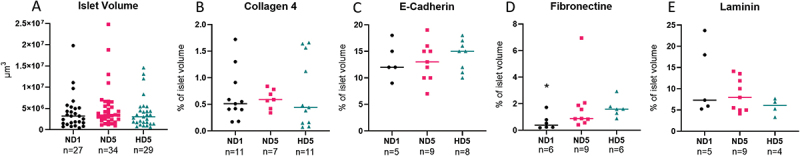
Summary of quantifications of protein content in human islets using DIA. Each dot in the graph represents an islet, and horizontal lines represent the mean value of the set. Statistical significance is shown with a *. (a) Islet volume did not differ significantly amongst groups. (b – e) target protein abundance expressed as a percentage of total islet volume. Means do not differ significantly, especially after 5 days of culture (*p* > .05).

Quantifications were performed using ad-hoc protocols created with Imaris® software, as illustrated in Figure 5. From a volumetric point of view, the mean amounts of studied proteins were not significantly different among the different conditions (Figure 6).

Taken together, these results show that hypoxia does not affect Collagen 4, Fibronectin, Laminin, and E-Cadherin overall content.

### 3D qualitative morphological analysis of islet ECM components

Volumetric quantifications fail to grasp the complexity of islet ECM. To better understand the distribution of the studied proteins, we analyzed images qualitatively, using a systematic approach to evaluate islets morphologically.

**Figure 7.**
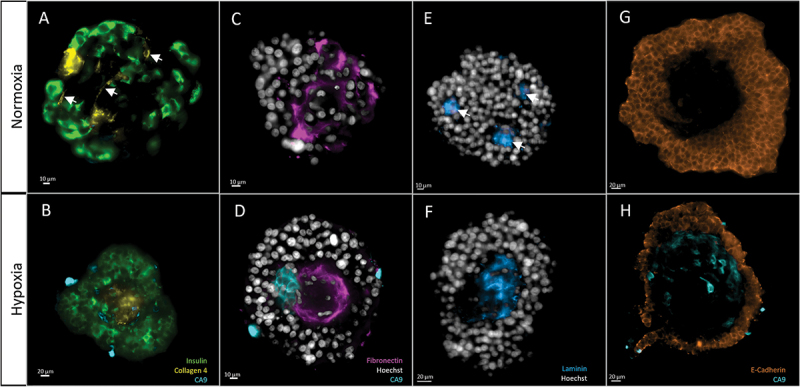
Morphological analysis of target proteins in human islets, after 5 days of either normoxia (top row) or hypoxia (bottom row). 2D cross sections in the middle of the islets. The same display parameters are identical for each protein. (a) Collagen 4 can be found in structures resembling blood vessels in group ND5 (arrows). (b) Central scar showing condensed Collagen 4 stain; note hypoxic cells (CA9-positive) around this area. (c) Fibronectin is found throughout the islet in normoxic conditions. (d) In hypoxia, it accumulates in the center of the islet, in the fashion of scarring tissue. This last picture is also a clear example of what we name architectural loss: ECM condensation, and scarce nuclei, which also present altered morphology, like irregular shape and small size. Note the presence of hypoxic cells (CA9) around the fibrotic core. (e) Laminin forms small, scattered nodes in normoxic conditions. (f) In hypoxia, it is found in central scars. (g) E-cadherin, a membrane marker, is found in the outer layers of islets while avoiding central areas. H) these holes in the signal are more evident in hypoxic conditions, where the central area can be populated by hypoxic cells (CA9-positive).

All islets were deprived of their basal membrane: we could not identify ECM proteins surrounding islets. We identified ECM inside the islet, although its distribution varied among groups. These morphological differences in the distribution of target proteins are summarized in Figure 7.

Collagen 4 was found around structures resembling blood vessels in the ND1 and ND5 groups, being predominant in the latter. In HD5, vessel-shaped structures could not be identified. Also, Collagen 4 was found in scarring areas in all groups, although these were more prominent in HD5, where they occupied large areas in the center of the islets.

Fibronectin was present throughout the islet. Outside the areas of scarring, it had a characteristic intercellular distribution. This mesh-like structure was more evident at ND5. In HD5, however, fibronectin tended to accumulate in central scars, surrounded by hypoxic areas.

Laminin, like the other ECM proteins, tended to accumulate in scarring areas. These areas were evident in HD5, presenting the central scarring already described. However, in ND5 these areas were smaller and scattered throughout the islet. We named these laminin-stained areas nodes. We found that the mean number of nodes was significantly lower in HD5, compared to ND1 and ND5 (respectively 1.5, SD 0.5, *n* = 4, vs. 6.4, SD 0.6, *n* = 5, and 7, SD 1.8, *n* = 9 *p* < .05) (Extended Data – Figure 3). This observation suggests that hypoxic conditions induce extensive central scarring, while in normoxia, the scarring is present but circumscribed to smaller but more numerous, scattered areas. Of note, node number and islet volume were correlated (PCC = 0.864, *p* < .01).

Taken together, the morphological analysis shows that ECM proteins (Collagen 4, Fibronectin, and Laminin) tended to accumulate in central areas of scarring tissue, suggesting central necrosis, which was more evident after 5 days of culture, especially in the hypoxia group. These areas were characterized by (1) nuclei with hyperintense signal, scattered and with aberrant shape, and (2) accumulation of ECM proteins presenting high density and a disorganized morphology (Figure 7b,d,f).

E-Cadherin presented a typical membrane distribution. In the ND1 group, staining was homogeneous, with only some sporadic areas of higher intensity. The islets from the ND5 group presented some areas of more intense staining, spatially related to the few zones stained for CA9. However, islets at HD5 presented many areas of high signal intensity, with an overall mean intensity significantly higher than other groups (ND1 = 8598, SD 1318, *n* = 5, ND5 = 6463, SD 657, *n* = 9, vs HD5 = 14805, SD 1762, *n* = 8, arbitrary units, *p* < .01) (Extended Data – Figure S3).

ND5 and HD5 were characterized by the presence of central holes, which correlated with islet size (PCC = 0.887, *p* < .01). When hole size was expressed as a percentage of total volume, we identified a tendency for bigger holes in HD5, although not statistically significant (2.5%, SD 2.5, *n* = 5, 3.1%, SD 1.3, *n* = 9, and 8.6%, SD 4, *n* = 8, for ND1, ND5, and HD5, respectively) (Extended Data – Figure s3).

Taken together, our results show the feasibility of volumetric quantification of Collagen 4, Fibronectin, Laminin, and E-Cadherin. In addition, the 3D approach for morphological assessment provides deep insights into protein distribution, exposing differences among experimental conditions that cannot be grasped with mere total protein quantification or 2D analysis.

## Discussion

This study is a proof of concept, demonstrating the applicability of a methodology for 3D imaging and analysis. Our protocol permitted the assessment of specific proteins in isolated pancreatic islets, both quantitatively and qualitatively. We have applied this methodology to evaluate the impact of hypoxia on E-Cadherin and three proteins of the extracellular matrix, namely Collagen 4, Fibronectin, and Laminin. To the best of our knowledge, we are the first to propose a complete protocol using a 3D approach for IF, clearing, LSFM, and DIA applied to rat and human, isolated pancreatic islets.

Our results support the validation of the immunofluorescence (IF) protocol, both for the absence of antibody cross-reaction and for homogeneous antibody penetration. To ensure diffusion, several factors needed to be optimized, notably pre-permeabilization, antibody concentration, buffer composition, and incubation time and temperature.^36^ Here, we have successfully applied it to both human and rat islets.

During validation, our results showed that when compared to a standard measurement method, i.e., radioimmunoassay, the DIA approach can similarly highlight differences in insulin and glucagon expression. Although meant as a validation step, our results open the door to a significant improvement in protein analysis. LSFM can indeed provide information on individual islets instead of the pulled measurements obtained by RIA or other proteomics quantification methods. It also provides information on protein distribution within each islet.

Following ECM loss during isolation, islets have been shown to undergo ECM remodeling.^11,32^ Nevertheless, the mechanisms underlying this phenomenon in the context of hypoxia remain largely unexplored.^9,32^ We propose here a technical approach to explore the complexity of the variations of ECM in hypoxic islets via qualitative and quantitative analyses.

The overall content of Collagen 4, Fibronectin, and Laminin, assessed by volumetric quantification, was not significantly affected by hypoxia. This can be understood from the point of view of energy consumption: any change in ECM after isolation involves an active process. In hypoxic conditions, the ability to secrete insulin is significantly reduced, as demonstrated by the glucose-stimulated insulin secretion (GSIS) test. Consequently, the remodeling of extracellular proteins may also be compromised, since this process relies on the secretion of enzymes. Furthermore, we have shown that through morphological analysis, only possible in a 3D setting, we could highlight subtle differences that could illustrate the influence of hypoxia on ECM. In other words, even if protein content is comparable, hypoxia induces a loss of architecture, resulting in a localized, central concentration of ECM proteins, creating scars inside the islet. We hypothesized that these areas are virtually lost and that the functional volume of the islet is thus reduced. In addition, the presence of hypoxic cells around necrotic cores could imply the presence of transitioning areas, where the rescue of dying cells could be a therapeutic target.

We observed that the E-Cadherin signal was absent in the central areas of the islet. As these central areas are occupied by an accumulation of ECM in other IF combinations, we hypothesized that holes correspond to areas of central scarring. Reports have shown an enhanced expression of E-Cadherin where ECM is absent and vice versa, suggesting compensatory mechanisms^12^.

These advances can support the development of new strategies to improve islet transplantation. For example, as many groups have proposed the use of ECM proteins and moieties to enhance survival and function,^10,13,16,19–24^ it would be interesting to explore their impact on the 3D structure of islets. Moreover, our technique could be applied to examine islets *ex vivo* to assess the impact of support techniques such as encapsulation or the use of biomaterials, and to evaluate islet morphology at different stages after transplantation.

Nevertheless, the described methodology has its limitations. LSFM remains an onerous technology, although it has become more common over the years.^37^ Also, our LSFM has only four excitation channels, limiting the number of concomitant IF stains. Furthermore, the selection of the antibodies can be challenging, as they need to come from different species and be sufficiently specific to avoid cross-reactions, limiting the possible combinations of stains.

Another limitation is the length of the protocol. For human islets, it takes 10 days from fixation to DIA results. However, this length is determined mainly by antibody incubation times; in our experience, this could still be reduced as the protocols evolve and are optimized. Furthermore, standardization could include the automatization of DIA, reducing analysis time and enhancing data quality.^42^

Albeit these surmountable limitations, we have developed a robust, validated methodology that could prove a major tool in a rapidly evolving field.^5,43^ As nowadays most quantification tools available cannot grasp morphology or spatial distribution, we believe that our 3D approach can play an important role in bettering the comprehension of isolated islets’ anatomy. Furthermore, our contributions to the understanding of ECM morphology could have a direct impact on improving therapeutic approaches.

## Conclusions

In this study, we have applied an incipient yet innovative technology to the study of pancreatic islets. With our 3D evaluation of ECM proteins and E-cadherin, we shed new light on the morphology of islets and demonstrated changes developed during hypoxia. Our findings fill a theoretical gap in the understanding of the remodeling of ECM after islet isolation, from a morphological perspective.

## Supplementary Material

Supplemental Material

## Data Availability

Images, including individual metadata and performed analyses, are available and free for use at https://doi.org/10.14428/DVN/GVARMX

## List of abbreviations

T1D: type 1 diabetes
ECM: extracellular matrix
BM: basal membrane
LSFM: light sheet fluorescence microscopy
IF: immunofluorescence
DIA: digital image analysis
RIA: radioimmunoassay
CA9: carbonic anhydrase 9
ND1: Normoxia Day 1 (study group)
ND5: Normoxia Day 5 (study group)
HD5: hypoxia Day 5 (study group)
CUBIC: clear, unobstructed brain/body imaging cocktails and computational analysis
