# Supplementary material for: 3D evaluation of the extracellular matrix of hypoxic pancreatic islets using light sheet fluorescence microscopy
Source: Islets. 2024 Jan 24;16(1):2298518. doi: 10.1080/19382014.2023.2298518 (PMC10810165; doi:10.1080/19382014.2023.2298518)
Supplement: Supplemental Material [file KISL_A_2298518_SM0064.zip › Extended data_DIA protocols clean.docx]

**Algorithm for ROI creation, using Imaris, as produced using Surface creator.**

Parameters are adapted to individual islets, using combined channels, but applying always the same criteria, to obtain a homogeneous ROI (no internal holes) without altering the islet size.

Highlighted: parameters to modify and adapt.

**Surfaces Creation Parameters**

  Format Version: **9.8**

  Enable Region Of Interest: **false**

  Process Entire Image: **false**

  Enable Region Growing: **false**

  Enable Tracking: **false**

  Enable Classify: **false**

  Source Image Index: **0**

  Source Channel Index: **0**

  Enable Smooth: **true**

  Surface Grain Size: **5** – minimum blur possible to avoid intra-ROI holes, while avoiding changes in total size

  Enable Eliminate Background: **false**

  Diameter Of Largest Sphere: **1.71263**

  Enable Automatic Threshold: **true**

  Manual Threshold Value: **1269.98** – minimum value possible to avoid intra-ROI holes, while avoiding changes in total size

  Active Threshold: **true**

  Enable Automatic Threshold B: **true**

  Manual Threshold Value B: **7410.05**

  Active Threshold B: **false**

  Region Growing Estimated Diameter: **2.28351**

  Region Growing Background Subtraction: **true**

  Enable Shortest Distance: **false**

  Surfaces Base Color: **1 1 1**

  Track Base Color: **1 1 1**

**Region Of Interest Container**

**Region Of Interest**

      Min X Y Z: **0 0 0**

      Max X Y Z: **1919 1919 159**

      Min T: **0**

      Max T: **0**

      Name: **Region of Interest 1**

      Active: **true**

      Selected: **false**

**Region Growing Filter**

**Statistics Filter**

      Lower Threshold Enable: **true**

      Lower Threshold Manual: **false**

      Lower Threshold Manual Init To Auto: **true**

      Lower Threshold Manual Value: **0**

      Upper Threshold Enable: **false**

      Upper Threshold Manual: **false**

      Upper Threshold Manual Init To Auto: **true**

      Upper Threshold Manual Value: **1**

      Select High: **true**

      Manual Threshold: **false**

      Manual Threshold Value: **0.000**

      Init Manual Threshold To Auto: **true**

**Statistics Value Type**

        Name: **Quality**

        Unit:

        Factors: **0**

**Surfaces Filter**

**Statistics Filter**

      Lower Threshold Enable: **true**

      Lower Threshold Manual: **true**

      Lower Threshold Manual Init To Auto: **false**

      Lower Threshold Manual Value: **10**

      Upper Threshold Enable: **false**

      Upper Threshold Manual: **false**

      Upper Threshold Manual Init To Auto: **false**

      Upper Threshold Manual Value: **1**

      Select High: **true**

      Manual Threshold: **true**

      Manual Threshold Value: **10.000**

      Init Manual Threshold To Auto: **false**

**Statistics Value Type**

        Name: **Number of Voxels**

        Unit:

        Factors: **1**

        Factor Name0: **Image**

        Factor Level0: **Image 1**

**Object Tracking Algorithm Parameters**

    Track Algo Name: **Autoregressive Motion**

    Fill Gap Enable: **false**

    Reference Frames Id: **0**

**Object Tracking Algorithm Linear Asignment**

      Max Gap Size: **3**

      Max Distance: **-1**

**Track Filter**

**Statistics Filter**

      Lower Threshold Enable: **true**

      Lower Threshold Manual: **true**

      Lower Threshold Manual Init To Auto: **false**

      Lower Threshold Manual Value: **2.5**

      Upper Threshold Enable: **false**

      Upper Threshold Manual: **false**

      Upper Threshold Manual Init To Auto: **true**

      Upper Threshold Manual Value: **1**

      Select High: **true**

      Manual Threshold: **true**

      Manual Threshold Value: **2.500**

      Init Manual Threshold To Auto: **false**

**Statistics Value Type**

        Name: **Track Duration Steps**

        Unit:

        Factors: **0**

**Surfaces Classification Parameters**

    Category: **Surface**

**Surfaces Event Parameters**

    Category: **Surface**

**Algorithm for the quantification of target proteins, using Imaris, as produced using Surface creator.**

Parameters are adapted to individual protein characteristics, but applying always the same criteria: to eliminate background noise, without cut-off in maximum signal. Each protein (insulin, collagen, etc.) require a specific threshold. The same threshold is applied to the entire batch.

Highlighted: parameters to modify and adapt.

**Surfaces Creation Parameters**

  Format Version: **9.8**

  Enable Region Of Interest: **false**

  Process Entire Image: **false**

  Enable Region Growing: **false**

  Enable Tracking: **false**

  Enable Classify: **false**

  Source Image Index: **0**

  Source Channel Index: **2**

  Enable Smooth: **true**

  Surface Grain Size: **0.456701**

  Enable Eliminate Background: **false**

  Diameter Of Largest Sphere: **1.71263**

  Enable Automatic Threshold: **false**

  Manual Threshold Value: **1855.94** – note: deletion of background noise

  Active Threshold: **true**

  Enable Automatic Threshold B: **true**

  Manual Threshold Value B: **65329.2** – note: this is the maximum value available

  Active Threshold B: **false**

  Region Growing Estimated Diameter: **2.28351**

  Region Growing Background Subtraction: **true**

  Enable Shortest Distance: **false**

  Surfaces Base Color: **1 1 1**

  Track Base Color: **1 1 1**

**Region Of Interest Container**

**Region Of Interest**

      Min X Y Z: **0 0 0**

      Max X Y Z: **1919 1919 159**

      Min T: **0**

      Max T: **0**

      Name: **Region of Interest 1**

      Active: **true**

      Selected: **false**

**Region Growing Filter**

**Statistics Filter**

      Lower Threshold Enable: **true**

      Lower Threshold Manual: **false**

      Lower Threshold Manual Init To Auto: **true**

      Lower Threshold Manual Value: **0**

      Upper Threshold Enable: **false**

      Upper Threshold Manual: **false**

      Upper Threshold Manual Init To Auto: **true**

      Upper Threshold Manual Value: **1**

      Select High: **true**

      Manual Threshold: **false**

      Manual Threshold Value: **0.000**

      Init Manual Threshold To Auto: **true**

**Statistics Value Type**

        Name: **Quality**

        Unit:

        Factors: **0**

**Surfaces Filter**

**Statistics Filter**

      Lower Threshold Enable: **true**

      Lower Threshold Manual: **true**

      Lower Threshold Manual Init To Auto: **false**

      Lower Threshold Manual Value: **10**

      Upper Threshold Enable: **false**

      Upper Threshold Manual: **false**

      Upper Threshold Manual Init To Auto: **false**

      Upper Threshold Manual Value: **1**

      Select High: **true**

      Manual Threshold: **true**

      Manual Threshold Value: **10.000**

      Init Manual Threshold To Auto: **false**

**Statistics Value Type**

        Name: **Number of Voxels**

        Unit:

        Factors: **1**

        Factor Name0: **Image**

        Factor Level0: **Image 1**

**Object Tracking Algorithm Parameters**

    Track Algo Name: **Autoregressive Motion**

    Fill Gap Enable: **false**

    Reference Frames Id: **0**

**Object Tracking Algorithm Linear Asignment**

      Max Gap Size: **3**

      Max Distance: **-1**

**Track Filter**

**Statistics Filter**

      Lower Threshold Enable: **true**

      Lower Threshold Manual: **true**

      Lower Threshold Manual Init To Auto: **false**

      Lower Threshold Manual Value: **2.5**

      Upper Threshold Enable: **false**

      Upper Threshold Manual: **false**

      Upper Threshold Manual Init To Auto: **true**

      Upper Threshold Manual Value: **1**

      Select High: **true**

      Manual Threshold: **true**

      Manual Threshold Value: **2.500**

      Init Manual Threshold To Auto: **false**

**Statistics Value Type**

        Name: **Track Duration Steps**

        Unit:

        Factors: **0**

**Surfaces Classification Parameters**

    Category: **Surface**

**Surfaces Event Parameters**

    Category: **Surface**
