# Supplementary figures and images for: 3D evaluation of the extracellular matrix of hypoxic pancreatic islets using light sheet fluorescence microscopy
Source: Islets. 2024 Jan 24;16(1):2298518. doi: 10.1080/19382014.2023.2298518 (PMC10810165; doi:10.1080/19382014.2023.2298518)

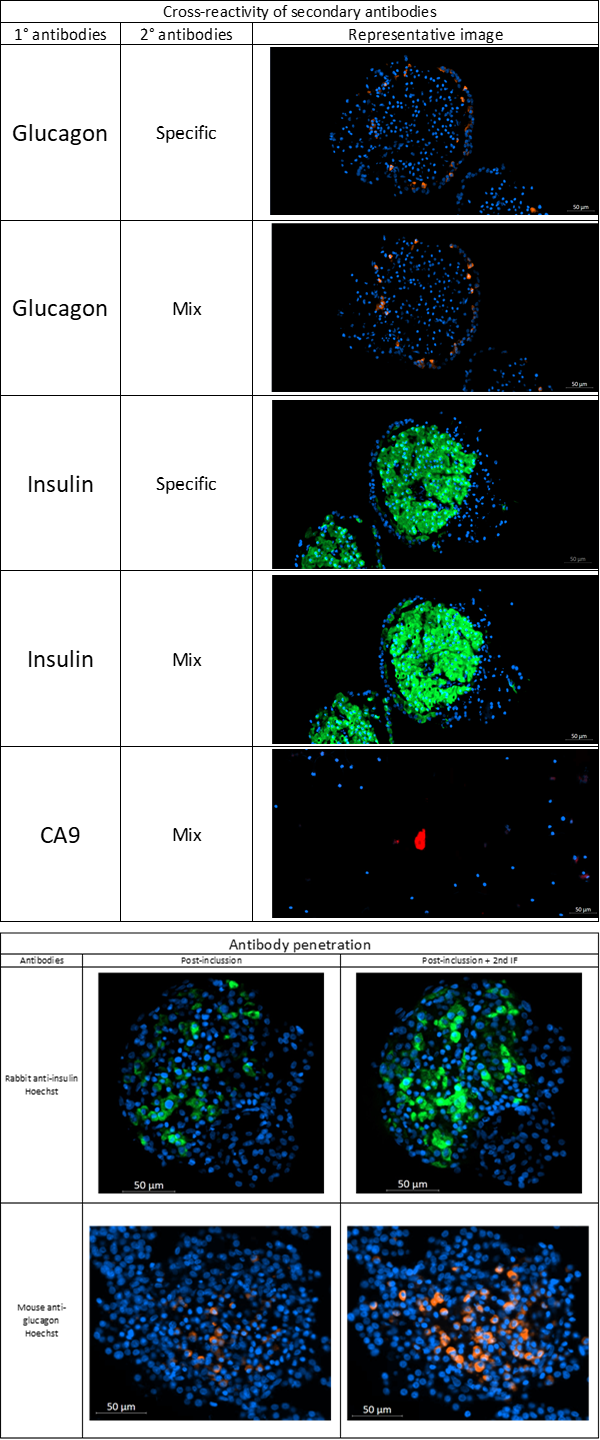

Supplement: Supplemental Material [file KISL_A_2298518_SM0064.zip › Extended data Figure 1.PNG]

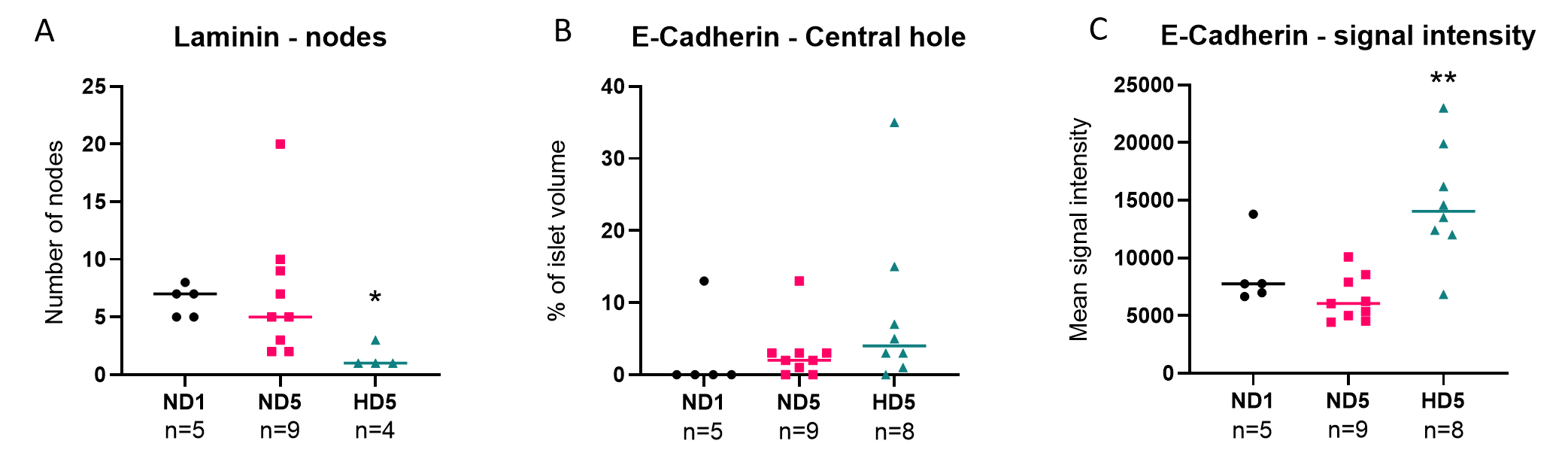

Supplement: Supplemental Material [file KISL_A_2298518_SM0064.zip › Extended data_Figure 3.PNG]

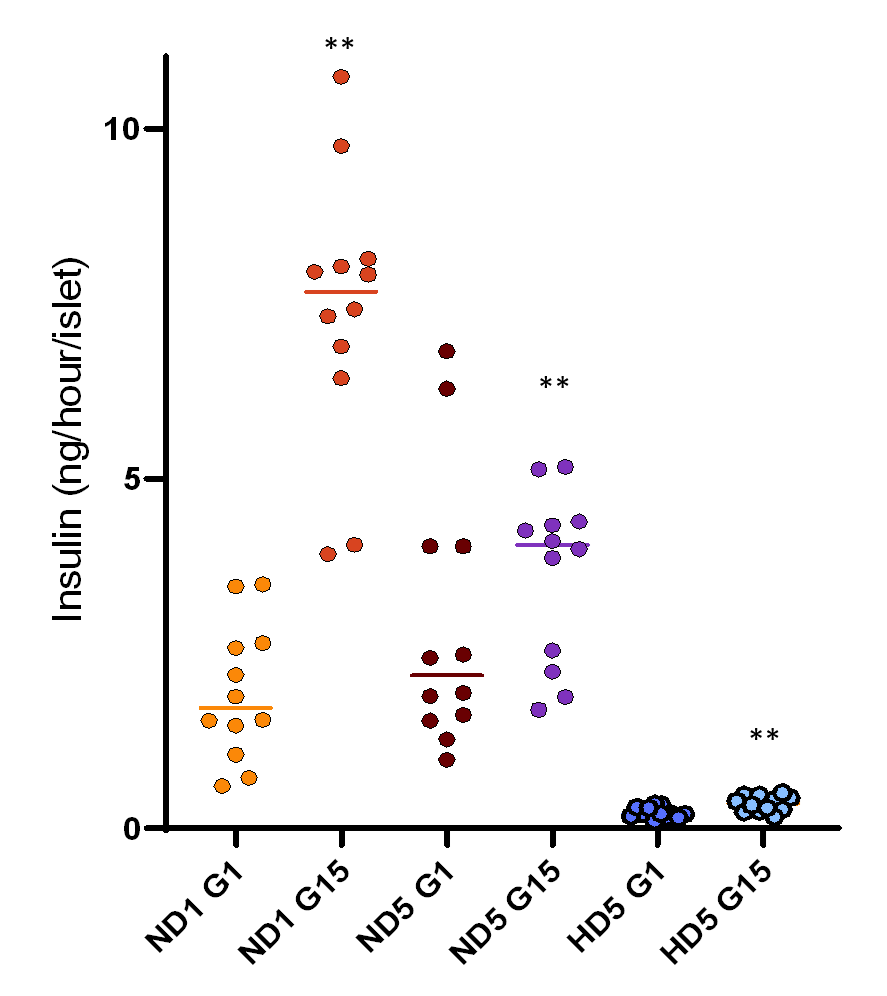

Supplement: Supplemental Material [file KISL_A_2298518_SM0064.zip › Extended data_Figure 2.PNG]
